# Parent's Attitudes toward Their Children's Oral Health Care during the COVID-19 Pandemic: A Cross-Sectional Study

**DOI:** 10.1155/2023/7340105

**Published:** 2023-07-11

**Authors:** Mahdieh Zarabadipour, Aida Mokhlesi, Taniya Poorsoleiman, Monirsadat Mirzadeh

**Affiliations:** ^1^Department of Oral and Maxillofacial Medicine, Dental Caries Prevention Research Center, Qazvin University of Medical Sciences, Qazvin, Iran; ^2^USERN Office, Qazvin University of Medical Sciences, Qazvin, Iran; ^3^Student Research Committee, Qazvin University of Medical Sciences, Qazvin, Iran; ^4^Community Medicine, Metabolic Diseases Research Center, Research Institute for Prevention of Non-Communicable Diseases, Qazvin University of Medical Sciences, Qazvin, Iran

## Abstract

**Background:**

The COVID-19 pandemic has significantly impacted global health and presented challenges to maintaining oral health in children. Efficient management and prevention of oral diseases are therefore crucial in this population.

**Aim:**

This cross-sectional study aimed to assess parents' self-reported oral and dental hygiene habits for their children during the COVID-19 pandemic.

**Methods:**

A total of 256 parents of primary school children in Qazvin completed an online questionnaire containing demographic, general, and cardinal questions. The collected data were analyzed using SPSS 22 software, and accurate analytical tests were run to reach the results.

**Results:**

The study found that 69.1% of parents and 87.1% of children brushed their teeth regularly during the pandemic. Additionally, 80.5% of parents monitored their children while brushing. While some parents expressed concerns about COVID-19 infection, parents from lower socioeconomic status reported a higher willingness to attend dental centers than those from more prosperous areas.

**Conclusion:**

These findings suggest that parents' supervision and attitude toward oral health care significantly affect children's oral hygiene habits during the pandemic, possibly due to increased time spent together during lockdowns.

## 1. Introduction

The COVID-19 pandemic has significantly impacted daily life worldwide, particularly in healthcare systems [1, 2]. While much of the focus has been on the respiratory effects of the virus, there needs to be more attention paid to preventive healthcare for children [3, 4]. Many parents are understandably concerned about their children's oral health, as regular dental care access may be limited in some areas [5, 6]. Maintaining good oral health from a young age is crucial to prevent dental decay or periodontal problems throughout life [7].

Parents play a critical role in ensuring their child's oral health care routine, starting from a young age [5]. They should advise their children to be cautious of what they put in their mouths and implement good dental hygiene practices [5].

A child's dental development is a long-term process, and each stage has a unique significance. A healthy mouth not only means a child's mouth is free from dental cavities or other issues but also represents their overall health in the long term [8]. However, the pandemic has significantly impacted access to preventive care services to maintain good oral health, which can negatively affect a child's dental health care [5, 9].

Moreover, given the nature of dental care, which involves close examination and working near the mouth, there are concerns about the risk of infection and transmission of the virus in dental care settings [8, 10]. As a result, many dental practices have suspended or reduced nonurgent clinical care services to protect patients and dental care professionals [11]. This action has created an additional challenge in caring for a child's oral health [12].

Parents may be worried about their children's oral health during the pandemic and may question how to maintain it amid restrictions on dental care provision [13]. The pandemic has introduced disruptions and challenges for healthcare providers and consumers, including dentists and parents [13].

This cross-sectional study explores parents' attitudes and behaviors toward dental care for their children during the COVID-19 pandemic, identifying potential focus areas for future preventive care.

## 2. Materials and Methods

### 2.1. Study Design

**Figure 1:**
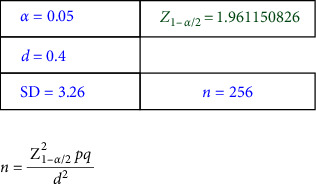
Sample size.

This analytical cross-sectional study involved parents of primary school children aged 7–12 from Qazvin in April and May 2021. To ensure a representative sample size, we estimated 256 participants with a 95% confidence level and a 5% margin of error (Figure 1). After obtaining approval from the Ministry of Education and Training, we drew a classified random sample from two regions of the city (north and south) based on economic and social factors. Two schools from each area (one boy and one girl) were selected using convenience sampling, and parents of the selected schools were then sampled.

### 2.2. Questionnaire Design

The questionnaire consisted of 50 questions divided into three sections: demographic [13], general [7], and cardinal [14], designed to assess the study objectives. The demographic section included information on the child and parent, such as gender, age, total family income level, parent's level of education, and parent's occupation. The general section aimed to determine factors influencing oral health in parents and children, while the specific area evaluated oral healthcare habits and the frequency of dental center visits during and before the COVID-19 pandemic (Table 1). The questionnaire's validity was approved by pediatric dentists, public health dentistst, and psychiatrists (correlation 95%). Reliability testing involved 20 participants and achieved an acceptable Cronbach's *α* value of 76%. The questionnaire was then made available online from April to May 2021.

### 2.3. Statistical Analyses

Data obtained from the questionnaire were analyzed using IBM-SPSS 22.0 software, with Crosstabs, *χ*^2^, and *t*-tests used for statistical analysis. Categorical data were expressed as frequency and percentage, while continuous data were described as mean ± standard deviation. *p* < 0.05 was considered significant.

### 2.4. Ethical Consideration

This study was approved by Qazvin University of Medical Science's research ethics committee (IR.QUMS.REC.1400.109), and all respondents gave informed consent before participating. The obtained data were only used for this study, and measures were taken to ensure the anonymity of the participants.

## 3. Results

### 3.1. Demographic Information

Two hundred fifty-six parents with an average age of 37.10 ± 5.480 years participated in this cross-sectional study. Most parents had a high school diploma; 76.2% of mothers were homemakers, while 43.8% of fathers were self-employed. Half of the families had two children, and 68% of parents believed their total family income level was low. The children's gender distribution was 1:1.28 (boys vs. girls) (Table 2).

### 3.2. General Information

Of the 256 parents, most (69.5%) reported that at least one family member (child or spouse) had contracted COVID-19. Nearly half (48.4%) agreed that the risk of contracting the virus in dental centers was similar to that in public places. While most parents (68.8%) were aware of the health protocols implemented by dental centers during the pandemic, a similar percentage reported feeling anxious and depressed during this period. More than 57% of parents noted an increase in their children's consumption of healthy foods (fruits and vegetables), although 75.8% indicated that the number of daily meals remained unchanged during the pandemic. There were no significant changes in parental supervision of children's dietary patterns, while the pandemic led to an increase in the overall food supply costs for 40.6% of parents.

### 3.3. Cardinal Information

During the pandemic, 69.1% of children consistently brushed their teeth compared to 64.5% before the pandemic, with a significant statistical difference observed (*p*-value = 0.00). Children from vulnerable areas had lower rates of consistent brushing compared to those of prosperous regions and those with diploma-holding and housewife mothers. Regarding parental behavior, 89.5% of parents brushed their teeth daily during the pandemic compared to 35.9%. The use of auxiliary health aids, such as dental floss and mouthwash, remained consistent before and during the pandemic, with no significant difference observed in parents' behavior compared to before (*p*-value = 0.00).

Regarding parental supervision of their children's brushing, 80.5% of parents monitored their child's brushing, with over half (52.7%) also helping their child. Parental supervision remained consistent before and during the pandemic, with higher rates among self-employed fathers and housewife mothers. A significant statistical difference was observed in supervision rates between vulnerable and prosperous areas, with higher rates reported in vulnerable regions (*p*-value = 0.023). Of those who said supervision changes, 86% had increased their monitoring. There was a significant statistical difference between parental supervision and regular brushing of children during both periods (*p*-value = 0.017 and 0.001, respectively).

Regarding dental center visits, only 25.7% of children visited a dental center during the pandemic, with vulnerable areas more likely to see it than prosperous areas (*p*-value = 0.038). Of those who stayed, most sought treatment for caries (44.6%). On the other hand, 60.5% of parents preferred their children not to visit dental centers, citing the possibility of contracting COVID-19 (36.3%) as the primary concern. Among parents open to taking their children to dental centers, a significant proportion were self-employed fathers (45.5%) and housewife mothers (84.2%) with diploma-level education. No significant difference was observed between parents' education level or occupation and their willingness to refer their children to dental centers. Vulnerable areas also had a higher desire for dental center visits during the pandemic. A significant statistical difference was observed between parents' and children's desires to visit dental centers during the pandemic (*p*-value = 0.01).

## 4. Discussion

COVID-19 has affected all aspects of billion lives such as finances, education, lifestyle, and most important of all healthcare needs [15] and has led to less medical and dental services during the quarantine due to fear of infection [16]. However, in this situation people's attitude and efficient healthcare are dependent to different factors like level of awareness, economic and social situation and service accessibility [17, 18]. Some of the challenges that we are facing at this time are children's oral hygienic habits and their attendance to dental centers during the quarantine which we brought up in this study.

According to the results of this study most parents declared they were sometimes anxious or depressed during the coronavirus pandemic. Khademian et al. [19] studied stress, anxiety, and depression during COVID-19 pandemic in Iran and detected signs of anxiety, stress, and depression. Also Zarabadipour et al. [20] studied factors affecting the stress caused by COVID-19 outbreak in community and medical staff and stated that medical staff in coronavirus section have the highest stress score which can be related to their occupation. In our study similar to Khademian et al. [19] vast majority of parents expressed they were depressed or anxious which can be affected by the fear of getting infected by coronavirus during the pandemic. Wong et al. [21] demonstrated that updated health-related information can lead to decreased levels of stress. Therefore, government and health policy makers must present updated and precise information in order to prevent the spread of false information.

As a result of our study, coronavirus outbreak has led to the mitigation in total income in most family. Also, most parents stated that expenses for providing food had increased stay at home, closure schools, and so many jobs led people to spend most of their time at home and eat more food.

One of the concerns during this pandemic can be gaining unhealthy dietary habits that can lead to an increase in dental and oral diseases in children [22]. However, findings of this study showed an increase in consumption of healthy foods including fruits and vegetables. Goswami et al. [13] reported that they made some changes in their children's diet to prevent caries. They reported a decrease in the amount of sugar, an increase in the amount of fiber, and consumption of fruits and vegetables, and an increase in the consumption of milk and cheese in their diet [13]. Also Campagnaro et al. [23] demonstrated that some parents altered their children's diet and chose healthier food while others increased the consumption of processed food. Another study performed in South Africa recorded that changes happening due to COVID-19 quarantine can be a tremendous thread regarding to food supply providence in low-income families [24]. A survey in India demonstrated that states with less prevalence of anemia and less population of underweight people have more rate of coronavirus infection [25]. These studies are in contrast with our results. Since a healthy diet is essential for proper immune system function [26], this can be the reason for increase in food consumption among families in Qazvin during quarantine. Also, consumption of healthy food can be a result of the state's climate. However, we should not underestimate the role of media and education in correcting dietary patterns.

Since most parents in our study declared they experienced anxiety and depression related to the pandemic it can be concluded that they were more precise on their children's diet in order to decrease the needs of medical and dental treatments.

Parents participated in our study stated that their children brushed their teeth regularly before and during the pandemic. Considering socioeconomic status, children were mostly from prosperous area families than vulnerable area one. A lot of children who brushed their teeth regularly had housewife mothers which showed a statistically significant difference in comparison to other children (*p*-value = 0.03). This can be the result of mothers' presence in the house in order to supervise children's oral health care.

In this study most parents and children brushed their teeth once a day which was statistically more during the pandemic than before. Also, most parents stated that their family used different oral healthcare habitudes. According to other studies, parents' oral health care is directly associated with the number of their children's decayed teeth [14, 15, 27, 28]. Children whose parents controlled their brushing and sugar consumption had favorable dental and oral hygienic habits [29], which seems to be the result of parents' attitude toward children's oral health condition.

Norms and culture can affect many social factors like values, beliefs, protocols, and also children's oral health [30, 31]. According to some studies key elements including attitudes toward parents' oral health, general knowledge, and health status are very effective on children's oral health [32, 33]. When these elements were compared, parents' behaviors were far more related to children's behaviors than parents' knowledge and attitude. Therefore, these findings confirmed that children learn from their parents' behavior [34]. It has been widely shown that parents' characteristics such as knowledge, attitudes, and socioeconomic status affect children's oral health behaviors [34]. Our study demonstrated this association as well since there was statistically significant difference between parents and children's brushing their teeth regularly during the pandemic.

Also, our study demonstrated that most parents monitored their children's oral hygienic habits during the pandemic and most of them helped their children while brushing their teeth. Goswami et al. [13] studied parents' proceeds and attitudes toward their children's oral healthcare during the coronavirus 2019 pandemic and expressed that the mean score of parents afford was low. Lockdowns led many parents to pursue their jobs online from home and spend more time with their children [35]. This could be a reason for parents to have more supervision. Also, we found a significant relationship between parents' supervision and children's regular brushing which endorsed Barenie et al. [36] study that stated most mothers (71%) are aware of daily surveillance but almost 40% of them do so. Also, only 40% of children considered their oral health care. These findings emphasize on parents' important role in maintaining children's oral health habits.

Despite more attention paid to children's oral health, most parents declared their reason for visiting a dentist during the pandemic, was to treat tooth decay. By assessing the results most parents were not willing to go to dental centers for their children's or their treatment procedures and most of them marked possible coronavirus infection as their reason. Decreasing the possible risk of infection can also be the reason for fewer visits for checkups than therapeutic procedures. The decline in household income can be another reason for low attendance. Karacin et al. [37] stated that people may avoid medical treatments for serious health problems shortly due to fear of infection. Similar findings were observed in Campagnaro et al. [23] study. Some of their participants announced they did not leave their houses for dental or medical appointments. This brings up the fact that most people interfere with their own medicine instead of directly consulting a doctor. Fux-Noy et al. [38] studied pediatric dentistry procedures performed in several dental centers in Israel and found that people are less willing to go to dental centers for emergencies during a pandemic in comparison to before. Wong et al. [39] demonstrated that not only this pandemic has affected dental procedures but also it has influenced medical emergencies since most people avoided going to emergency centers due to the fear of confrontation with infectious people. Guo et al. [40] showed a 38% decrease in dental centers referral at the beginning of the pandemic. Also, another reason for the decrease in attendance and this unwillingness can be wrong notices and people's unawareness of hygienic protocols regarding patients' reception (including a number disinfection of equipment and surfaces and increased time between visits). However, since no precise questions were asked regarding people's willingness to go to dental centers before the pandemic, we can not present reasonable answers for parents' unwillingness to refer to dental centers.

## 5. Conclusion

According to the results of our study, during the COVID-19 pandemic, some jobs are closed or part-time, so parents spend more time at home because of this health-threatening disease. For this reason, their supervision of their child's oral care and family diet became increasingly important. In contrast, parents stated they were anxious about needing medical or dental treatment, so they tried to keep the family members healthier.

## 6. Highlights

1. A high percentage of supervising parents for the child's brushing and additional oral hygiene (dental floss and mouthwash) can be a reason for children to brush their teeth regularly during the COVID-19 pandemic. This can be due to parents spending more time with their children during lockdowns.
2. Most parents stated that the reason for the need for dental treatment was the treatment of their child's tooth decay during the COVID-19 epidemic due to the low attitude toward regular visits to the dentist during this period.
3. Most parents stated that the reason for not wanting to visit a dentist during the COVID-19 epidemic was the transmission of the COVID-19 virus during this period. However, most parents were familiar with the health protocols followed in dental centers during this corona epidemic.

## 7. Importance of the Study

1. The study highlights the need to raise awareness among parents about the importance of maintaining their children's oral health by implementing various oral hygiene measures.
2. There is a need to raise awareness about the various causes of dental caries and the importance of nutritional counseling, including controlled sugar consumption and high fiber and fruit in the diet.
3. It was noted that oral health education among parents, especially those from the lower socioeconomic strata, is low and needs to be increased.
4. There is a need to improve the availability of emergency dental services in hospitals, clinics, and outpatient clinics at the government and private levels, as many parents cannot access these services in times of need.
5. There is an urgent need to improve the concept and practice of teledentistry as it is helpful for future prevention and preparedness in the event of a health crisis such as the COVID-19 pandemic.

## 8. Limitations of the Study

1. It is better to use the DMFT index in future studies to measure the oral health status of children, as due to the closure of schools, we could not measure the DMFT index of children and report it in our study.
2. In future studies, it is better to use all the regions of Qazvin city for sampling the survey according to an indicator of population dispersion of the statistical population; due to the limited access to parents due to the closure of schools, this sampling method was not possible in this study.
3. Because academic dental centers were closed during the data collection period, we were unable to assess the participants' gingival and periodontal health in this study.

## 9. Implication for Practice Statement

COVID-19 has had a significant impact on global health and has affected people all over the world in various ways. So, effective management and prevention of the virus is crucial, especially concerning pediatric health.

## Figures and Tables

**Table 1:** Translated questionnaire.

Question	Category
(1) Your feelings (parent) during the outbreak of the coronavirus in Iran:	Generally anxious and depressed
	Sometimes anxious, sometimes depressed
	Generally hopeful

(2) Has anyone from your family been infected with corona during the epidemic?	Yes
	No

(3) Your (parent) concerns about going to dental centers compared to going to public places during the 2019 coronavirus pandemic:	Dental centers have a higher risk of transmission of the 2019 coronavirus than public places
	Public places are more likely to transmit the 2019 coronavirus disease than dental centers
	The possible risk of transmission of the 2019 coronavirus disease is the same in dental centers and public places

(4) Is your family familiar with the health protocols that are followed in dental centers during this corona epidemic?	Yes
	No

(5) Which option shows the effect of the coronavirus epidemic on the overall income of your family?	The overall family income has decreased
	The total income of the family has not changed
	The overall income of the family has increased

(6) Has there been a change in the cost of food supply during the coronavirus epidemic?	Yes, the cost of consumption has decreased
	Yes, the cost of consumption has increased
	No, there has been no change in the consumption cost

(7) Has there been a change in the food pattern of your family (you, your wife, and children) during the coronavirus epidemic?	Carbohydrate consumption is increased
	The consumption of healthy foods (fruits and vegetables) has increased
	Food pattern has not changed

(8) Does your child brush his teeth regularly during the corona epidemic? If yes, please answer the following questions	Yes
	No

(9) How much does your child brush their teeth regularly during the corona epidemic?	More than once a day
	Once a day
	To be in important places

(10) Did your child brush his teeth regularly before the corona epidemic? If yes, please answer the following questions	Yes
	No

(11) How much did your child brush their teeth regularly before the corona epidemic?	More than once a day
	Once a day
	To be in important places

(12) Do you (parent) brush your teeth regularly during the corona epidemic? If yes, please answer the following questions	Yes
	No

(13) How much Do you (parent) brush your teeth regularly during the corona epidemic?	More than once a day
	Once a day
	To be in important places

(14) Did you brush your teeth regularly before the corona epidemic? If yes, please answer the following questions	Yes
	No

(15) How much did you brush your teeth regularly before the corona epidemic?	More than once a day
	Once a day
	To be in important places

(16) Do your family use side hygiene aids (dental floss and mouthwash) in addition to brushing your teeth during the corona epidemic?	Yes
	No

(17) Did you and your family use side hygiene aids (dental floss and mouthwash) in addition to brushing your teeth before the corona epidemic?	Yes
	No

(18) Do you supervise your child's brushing? If yes, please answer the following questions	Yes
	No

(19) How do you supervise your child's brushing?	I monitor my child's brushing after finishing brushing
	I help my child to brush his teeth

(20) During the corona epidemic, has your monitoring of your child's oral and dental health changed? If yes, please answer the following questions	Yes
	No

(21) How has your monitoring of your child's oral and dental health changed?	Increase
	Decrease

(22) During the corona epidemic, has the frequency of your child's meals (main and side) changed? If yes, please answer the following questions	Yes
	No

(23) How has the frequency of your child's meals (main and side) changed during the corona epidemic?	Increase
	Decrease

(24) During the corona epidemic, has the amount of supervision you (parent) have on your child's eating pattern changed? If yes, please answer the following questions	Yes
	No

(25) How has the amount of supervision you (parent) have on your child's eating pattern changed?	Increase
	Decrease

(26) Have you noticed the presence of decay or a new cavity in your child's tooth during the corona epidemic?	Yes
	No

(27) Has your child visited a dentist during the corona epidemic? If yes, please answer the following questions	Yes
	No

(28) How has your child visited a dentist during the corona epidemic?	Perform orthodontic treatment
	Treat tooth decay
	Because of toothache
	Due to tooth trauma
	For a routine checkup

(29) Do you (the parent) want your child to visit dental centers?	Yes
	No

(30) If your answer to the q29 is yes, choose the reason for referring to dental centers	During the corona epidemic, I go to dental centers for my child's treatment
	During the corona epidemic, I go to dental centers for my child's emergency treatment

(31) If your answer to the q29 is no, choose the reason for not referring to the dental centers	There is a possibility of transmission of the 2019 coronavirus by visiting a dentist
	My child's dental treatment is not urgent or necessary Dental centers are closed
	Dental centers are closed
	Appointments with dentists were canceled by dental centers
	We canceled the appointment with the dentist (parents)

(32) Do you (your parent) go to the dentist during the corona epidemic? If yes, please answer the following questions	Yes
	No

(33) How do you (parent) go to the dentist during the corona epidemic?	Perform orthodontic treatment
	Treat tooth decay
	Because of toothache
	Due to tooth trauma
	For a routine checkup

(34) Do your (parent) want to go to dental centers?	Yes
	No

(35) If your answer to the q34 is yes, choose the reason for referring to dental centers	During the corona epidemic, I go to dental centers for my child's treatment
	During the corona epidemic, I go to dental centers for my child's emergency treatment

(36) If your answer to the q34 is no, choose the reason for not referring to the dental centers	There is a possibility of transmission of the 2019 coronavirus by visiting a dentist
	My child's dental treatment is not urgent or necessary Dental centers are closed
	Dental centers are closed
	Appointments with dentists were canceled by dental centers
	We canceled the appointment with the dentist (parents)

**Table 2:** Characteristics of the study participants.

Parameter	Categories	Frequency
Gender of children	Boy	112 (43.8)
	Girl	144 (56.2)

Gender of parent filling questionnaire	Male	25 (9.8)
	Female	231 (90.2)

Father education	Under diploma	54 (21.1)
	Diploma	94 (36.7)
	Bachelor's degree	68 (26.6)
	Master's degree	40 (15.6)

Mother education	Under diploma	41 (16.0)
	Diploma	116 (45.3)
	Under diploma	79 (30.9)
	Diploma	20 (7.8)

Father's job	Employee	195 (76.2)
	Self-employment	50 (19.5)
	Other	11 (4.3)

Mother's job	Housewife	195 (76.2)
	Employee	50 (19.5)
	Self-employment	11 (4.3)

Overall family income level	3–5 million tomans	174 (68)
	5–10 million tomans	58 (22.7)
	More than 10 million tomans	24 (9.4)

Social level	Prosperous areas	132 (51.6)
	Vulnerable areas	124 (48.4)

## Data Availability

The data used to support the findings of this study are included in the article.

## Abbreviations

COVID-19: Coronavirus 2019
M: Male
F: Female.
